# Provision of neuropsychiatry services: variability and unmet need

**DOI:** 10.1192/pb.bp.114.047324

**Published:** 2015-12

**Authors:** Niruj Agrawal, Rahul Bhattacharya, Hugh Rickards

**Affiliations:** 1 St George's Hospital, London; 2 Barts and the London School of Medicine and Dentistry; 3 University of Birmingham

## Abstract

**Aims and method**

Neuropsychiatry services remain underdeveloped and underprovided. Previous studies have shown variability in service provision in the UK. In this survey we approached all mental health and neuropsychiatric service providers within London to map current neuropsychiatric service provision and explore perceived barriers.

**Results**

All the specialist mental health service providers responded. There was huge variability in neuropsychiatric service provision within different parts of London. There was evidence of significant unmet need and variability in service pathways. Lack of earmarked funds for neuropsychiatry and disjointed funding stream for such services were identified by providers as a barrier.

**Clinical implications**

This study provides further evidence of an ongoing lack of adequate neuropsychiatric service provision. Reasons for variability and unmet need are discussed. Adoption of a previously proposed hub-and-spoke model of service provision and the removal of commissioning barriers through uniform national commissioning may help deal with this problem.

Neuropsychiatric conditions are frequently encountered in neuroscience settings and are not uncommon in mental health services. However, the provision and development of neuropsychiatric services has lagged behind in the UK and globally.^1,2^ Attempts at establishing the neuropsychiatric needs of the general population have taken two different routes. Researchers have either assessed ‘mental health needs’ in neurology patients or have searched for ‘organic’ problems in general psychiatric patients. A number of studies from Europe have estimated psychiatric morbidity in neurology patients at 40-55%. A study from London looking at neuroscience in-patients found a prevalence of neuropsychiatric conditions of 55%.^3^ Similar figures were reported from Scotland (47%) and additionally, a 30% prevalence of conditions ‘not explained by organic cause’ in neurology out-patients was described.^4^ A study from Scandinavia reported the prevalence of psychiatric conditions in neurology patients as 55.1%.^5^ However, referral rates to mental health services in this study were only 4.6%. Therefore, one should not assume that these relatively high needs are always addressed. Data on assessment of neurological or organic conditions in mental health patients are poor, but these are estimated to be around 10%.^6^

Estimates of the prevalence of neuropsychiatric presentations in specific neurological conditions range from 20 to 75%, depending on the nature and severity of the condition, method of assessment and the population studied.^7-9^ Rates of neuropsychiatric problems are generally higher in the specialist or tertiary centre settings.

What constitutes a neuropsychiatric condition and which of those conditions require specialist neuropsychiatric service input has also been open to interpretation. We have defined neuropsychiatric conditions under four broad categories in the accompanying paper.^10^ Not all patients in those categories would require specialist neuropsychiatric input. Neuropsychiatry services would see patients that are complex and beyond the service provision capacity of either neurology services or mental health services alone. Patients accessing neuropsychiatry services should have problems that fall within one of the four categories that define neuropsychiatric conditions^10^ and should meet at least one of the criteria described in Box 1.

Information on neuropsychiatry need and demand at a clinical services level is available from two large-scale audits carried out in south England, led by two of the most established providers of neuropsychiatric services in the UK.^11,12^ The authors concluded that geographical distance from a specialist service was the most significant barrier to access to care. They also suspected there was a lack of localised service provision for neuropsychiatry, both of which contributed to unmet need. Both studies found unmet neuropsychiatric need, particularly in areas geographically distant from neuropsychiatry centres. It was concluded that there was emerging consensus that a referral rate of less than 20 per 100 000 population per year possibly signified unmet need in terms of service provision.^6^

**Box 1** Criteria for referring a neuropsychiatric condition to a specialist neuropsychiatry service.

- Complex
- Requires specialist investigations
- Requires specialist assessment
- Requires specialist treatment
- Requires neuropsychiatric clinical expertise, which lies outside of that which may be expected in either mental health or neurology services.

Neuropsychiatric care pathways and commissioning are not standardised and are highly variable across the UK. We suspect that neuropsychiatry service provision and access to care for patients are likely to be influenced by other complex factors over and above the well-established ‘geographical distance’ from a centre of neuropsychiatry. In this study we aimed to explore whether there is still geographical variation in neuropsychiatry service usage. Rather than exploring this from a purely service provider perspective, we invited both service providers and service commissioners to share their views. Through this process we aimed to minimise potential bias of data on variability obtained in previous studies solely from tertiary referral centres. The ‘top-down’ approach of assessing variability purely from one specialist service provider perspective may be prone to bias as more than one provider may be catering to the needs of the population in an area and indeed some services may be provided locally outside large tertiary centres.

## Method

Two separate regional cross-sectional surveys were carried out. R.B. and N.A. devised two standardised questionnaires: one for commissioners and the other for service providers. The questionnaires were developed through iteration and in consultation with the national questionnaire survey into neuropsychiatry services.^14^ Questions were very broad and open ended to establish the current state of affairs rather than to prove or disprove an *a priori* hypothesis. Mapping of existing neuropsychiatry services in London was completed. Mapping of services was carried out by an electronic search for neuropsychiatric services by inputting words ‘neuropsychiatry’ and ‘London’ into a generic search engine. In addition, information on existing neuropsychiatry services was obtained through the Faculty of Neuropsychiatry at the Royal College of Psychiatrists and by calling all the major mental health trusts in the London area. All specialist mental health providers were identified within the target geographical area. National or tertiary level referral centres providing neuropsychiatry services and neurorehabilitation to the population of London, including public and private or independent providers, were also identified. Senior management for each provider were contacted electronically with the questionnaire and this was followed up by a telephone call.

All local mental health commissioners from primary care trusts (PCTs) were contacted using a similar method. The survey attempted to assess existing provision and service usage for neuropsychiatry to capture variation within the well-defined geographical units (boroughs covered by PCTs). We discovered there was a centralised regional (strategic health authority (SHA) level) specialist commissioning panel for neurorehabilitation in London that commissioned services related to acquired brain injury across the city. We approached it with the commissioning questionnaire for our survey. We also explored the commissioners' and providers' perceptions of neuropsychiatry and perceived barriers to neuropsychiatry service and commissioning. Data were verified and cross-checked between providers and commissioners, although it was recognised that commissioners often went to more than one provider for different elements of neuropsychiatry services. Data captured were subjected to descriptive analysis and no inferential statistics were used.

## Results

Data were collected from the local PCT commissioners and from the London-wide specialist commissioning group referred to above. Most local commissioners reported on commissioning that took place through generic mental health streams, as opposed to the specialist stream referred to by the specialist commissioning group. Mental health commissioners were requested to report actual figures, however, if these were not available they were asked to offer estimated figures based on the available data. There were 31 boroughs and 30 PCTs in London, which covered a population of 185 000 to 399 000 each (average 284 000). Response rate from the PCTs was very good (83%) and 100% responses were received from the providers of neuropsychiatric services in the area. We were not made aware of any patients going out of area from London for neuropsychiatric need, on the contrary, providers in London see a number of patients from outside the local area.

**Fig. 1:**
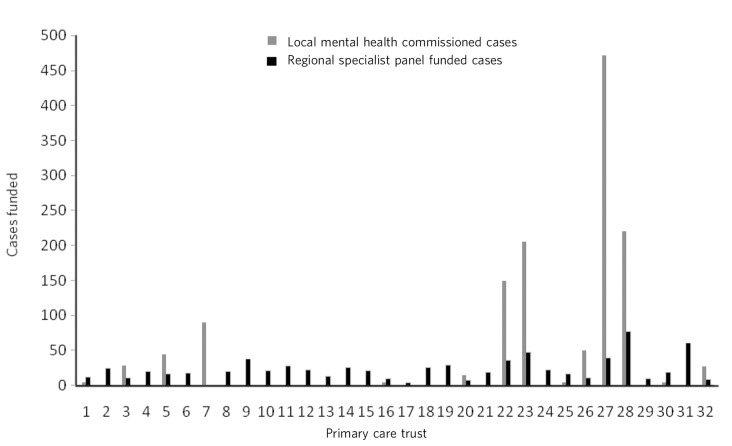
Breakup of neuropsychiatry cases funded.

The number of patients for the specialist commissioning group ranged from 3 to 76 per year (Fig. 1). Therefore, there was a 25-fold variation in incidence among the 30 referring geographical units within London. About half the PCTs (*n* = 15) were able to provide an estimate of the number of neuropsychiatry patients they funded: from 4 to 472 per year. The variation of incident referrals at the unit PCT mental health commissioning level was a staggering 118-fold. The variation in population between the 31 boroughs and the PCT catchment areas was approximately two-fold. In Fig. 1, bars 6 and 19 represent patients commissioned through specialist panels from two PCTs. For commissioning of neuropsychiatry through local commissioning they functioned as one unit and are represented as bar 7.

The commonest mode of commissioning of neuropsychiatry services was to tertiary services followed by local services. Funding streams for certain conditions were identified to be other than mental health, for example through acute care, physical health, neurosciences or older adults, or even Improving Access to Psychological Therapies (IAPT). Brain injury rehabilitation was commissioned through the pan-London specialist commissioning group referred to earlier. Certain services, which were not specifically commissioned, were provided by mental health trusts (therefore commissioning of these services remains unclear).

## Discussion

### Variability in service pathways

**Fig. 2:**
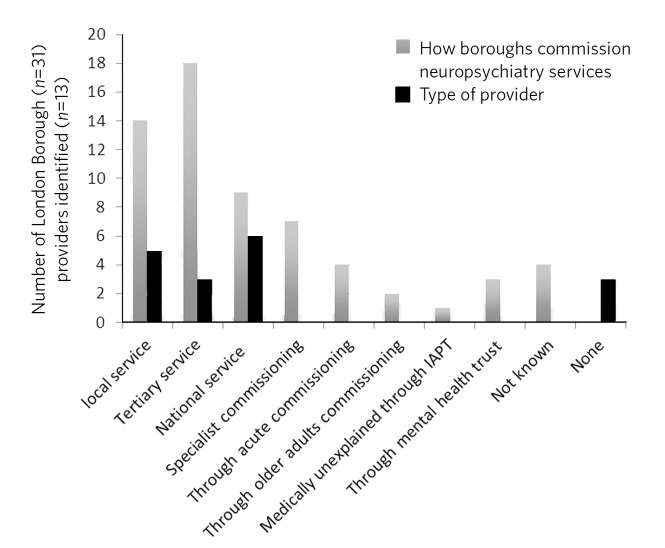
Variability in neuropsychiatric service provisions and commissioning. IAPT, Improving Access to Psychological Therapies.

Neuropsychiatry is a complex discipline which requires a highly skilled workforce dealing with a range of conditions. Different service models have been proposed to meet neuropsychiatric needs. In one of the models, neuropsychiatry services are based at a ‘tertiary level’, accepting referrals from psychiatry, neurology, geriatrics and general medicine.^15^ In London, though most neuropsychiatric service provision was at a tertiary level (Fig. 2), no clear service models or pathways of neuropsychiatry services emerge from the data. The lack of consistency of neuropsychiatry service provision in a relatively small geographical area is quite striking.

Internationally, different models for neuropsychiatry service provision have been reported. In Ireland, an in-patient neuropsychiatric service is closely aligned to neuroscience services and receives referrals from neurology and neurosurgery.^16^ Although neuropsychiatric services are commonly aligned to large neuroscience centres, successful neuropsychiatric services have been set up aligned to district or local general hospitals. There is a published report of such a service from the UK.^17^ Although there has been mention of community outreach model in line with stroke-related rehabilitation,^18^ there is little evidence that neuropsychiatry has adapted to such a service model. Most neuropsychiatry service models from outside the UK refer to a liaison consultation model.^10,19^ In the UK, the College's Faculty of Neuropsychiatry working group proposed a hub-and-spoke model, with the hub closely allied to the neurosciences centre but the spokes working closely with services in the community.^6^ Currently, there is no evidence that this model has yet been adopted consistently in London. There is an urgent need to create clear neuropsychiatry service pathways and a hub-and-spoke model is likely to be the best option.

### Continued unmet need in neuropsychiatry

This study found huge variability in annual rates of funded neuropsychiatry cases in the London area. This may to some degree represent poor data collection, or it may reflect real variability in the provision of neuropsychiatry services. The rate of referral in some areas was as low as 2 per 100 000 population, and estimates of referral below 20 per 100 000 have been proposed to represent an unmet local need for neuropsychiatry services.^6^ This study once again found evidence that there continues to be very significant unmet need in neuropsychiatry within London, despite the presence of a number of well-recognised neuropsychiatry services. There appears to be very little progress in meeting neuropsychiatric need in recent years.^11,12^ Barriers to commissioning, which may be responsible for the current state of affairs and are proving to be persistent, need to be explored.^10^

### Continued lack of equitable care

This survey continued to find significant variation in the number of patients accessing neuropsychiatry services in the different London areas served by different PCTs. Earlier audits by tertiary referral centres for neuropsychiatry had identified a significant variation in service usage from different areas. A south London study showed up to 34 times' variation in neuropsychiatry cases per 100 000 population, ranging from 0.910 to 30.8.^12^ A north London audit also discovered variation, although on a slightly smaller scale ranging from 1.7 to 25, which amounted to almost 15 times' variation between the highest and lowest referring boroughs or counties.^11^

Whereas these previous studies took a provider-facing approach, our study examined variation in service usage from both secondary and tertiary provider perspectives as well as local and specialist commissioner perspectives. It revealed a more acute variation in neuropsychiatry provision in London. There was approximately 25-fold variation across boroughs for head-injury-related admission for neuropsychiatric rehabilitation. This is similar to the variations noted above. This is in spite of the relatively homogeneous ‘caseness’ for acquired head injury, a relatively well-established service provision across London and the specialist commissioning panel dedicated to brain injury rehabilitation. Variability for out-patient neuropsychiatry provision was much more marked. The level of variability of provision in different areas of London cannot be explained by differences in demographics, which at best can explain a small degree of variation in a relatively small geographical area. This study shows that there is lack of equitable access to neuropsychiatry care in different parts of London that requires careful exploration and explanation.

### Reasons for variability and inadequate provision

This study concurs with the findings from the other two London studies that geographical distance from neuropsychiatry centres does adversely affect service usage. However, it indicates that there are other factors that contribute to this variability, given that the geographical distance from a centre of neuropsychiatric provision in London is not excessive.

We hypothesise that factors that present as barriers to care in neuropsychiatry include contractual arrangements, funding streams, awareness of neuropsychiatry among commissioners and providers, and national strategic drivers which have an impact on service provision. Areas local to tertiary or national neuropsychiatric services may have better communication with commissioners to overcome these barriers and more favourable contractual arrangements to minimise barriers to funding approval, compared with services located at a distance. Local mental health commissioners were more aware of neuropsychiatry as a discipline, its boundaries, funding streams and local needs when they were working in areas in close proximity to tertiary or national service provider. We also found that, in areas located in close proximity to neuropsychiatry centres, ‘secondary’ mental healthcare was sometimes provided by the same provider as the neuropsychiatry service, which may have minimised funding and pathway barriers.

### Limitations

The study was carried out within the area of Greater London, which may raise concerns about generalisability of the data to the rest of the UK. London traditionally has a better level of neuropsychiatry service provision and has well-known services that received referrals from outside London. Data from previous studies^11,12^ show that the provision of neuropsychiatry services outside London is not as good and the variability and unmet need is likely to be even more acute. Hence, the data from this study are pertinent to the whole of the UK and any solutions to deal with unmet need and variability should be applicable country wide. Indeed, given that a similar state of affairs has been reported anecdotally elsewhere in Europe,^5^ we believe the lessons learnt from this work are global.

The study looked into commissioning and provision from the mental health perspective and incorporated neurorehabilitation specialist commissioning. However, neuropsychiatry services are located at the interface of neurology and psychiatry and therefore the study may have failed to capture any neuropsychiatry service provisions that were embedded within acute healthcare setting. However, evidence of huge variability, unmet need and the fact that some boroughs had no local neuropsychiatric commissioning arrangements reasonably close to areas of neuropsychiatric service provision strongly suggests that provision for neuropsychiatry in London remains inequitable and inadequate.

The study surveyed service providers and commissioners and can only comment on the responders' understanding, knowledge and perception of how services were aligned, and provides proxy measures as opposed to real ones. The participants' responses might be affected owing to a lack of coherent understanding around caseness in neuropsychiatry. We have proposed a clearer definition of what constitutes a neuropsychiatric condition in Box 2 in the accompanying paper,^10^ and have defined the threshold criteria for when a referral should be made to a neuropsychiatric service for such conditions in Box 1 in this paper. In our opinion, a combination of a clear definition of neuropsychiatric condition and the threshold criteria will help resolve the issue of caseness.

This study provides further evidence of a continuing unmet need, significant variability of provision and lack of consistent service models and pathways in neuropsychiatry in the Greater London area. We believe this is representative of the situation in the rest of the UK, where the problem may be even worse given that London has a higher level of neuropsychiatric service provision with a few regional and national centres. The reasons for such variability need to be explored and minimised. Barriers to commissioning and provision^10^ need to be explored and removed. A hub-and-spoke model of neuropsychiatry provision closely allied with neurosciences centres^6^ should be adopted widely to bring consistency of pathways. National commissioning with a mandate for abolishing undesirable variability and unmet need is the real solution, but one that is not without significant challenges.
